# Privacy-Preserving Face Recognition Method Based on Randomization and Local Feature Learning

**DOI:** 10.3390/jimaging10030059

**Published:** 2024-02-28

**Authors:** Yanhua Huang, Zhendong Wu, Juan Chen, Hui Xiang

**Affiliations:** School of Cyberspace, Hangzhou Dianzi University, Hangzhou 310018, China; hyh1026@hdu.edu.cn (Y.H.);

**Keywords:** facial recognition, privacy protection, local randomization and learning, visual information elimination, privacy-preserving face recognition

## Abstract

Personal privacy protection has been extensively investigated. The privacy protection of face recognition applications combines face privacy protection with face recognition. Traditional face privacy-protection methods encrypt or perturb facial images for protection. However, the original facial images or parameters need to be restored during recognition. In this paper, it is found that faces can still be recognized correctly when only some of the high-order and local feature information from faces is retained, while the rest of the information is fuzzed. Based on this, a privacy-preserving face recognition method combining random convolution and self-learning batch normalization is proposed. This method generates a privacy-preserved scrambled facial image and an image fuzzy degree that is close to an encryption of the image. The server directly recognizes the scrambled facial image, and the recognition accuracy is equivalent to that of the normal facial image. The method ensures the revocability and irreversibility of the privacy preserving of faces at the same time. In this experiment, the proposed method is tested on the LFW, Celeba, and self-collected face datasets. On the three datasets, the proposed method outperforms the existing face privacy-preserving recognition methods in terms of face visual information elimination and recognition accuracy. The recognition accuracy is >99%, and the visual information elimination is close to an encryption effect.

## 1. Introduction

Due to the rapid development of technology and the use of large datasets, face recognition technology has been applied in various fields. However, the widespread use of face recognition systems brings some challenges, from which the issue of face privacy protection has been widely mentioned. Face recognition is closely related to property and personal safety. However, in order to rapidly promote face recognition technology, face databases without privacy protection are widely collected, which brings serious personal privacy security risks.

In a face recognition system, facial images are transferred to a server using an acquisition device and are compared with the stored facial feature database on the server. Servers mostly store clear and complete faces. Some PPTs (privacy-protection technologies) employ encryption and decryption methods, wherein the server-based facial feature database and the facial images are encrypted [1]. However, decrypting the incoming and the server-based facial images is necessary during recognition. Therefore, server-based databases encounter potential privacy breaches. Some PPTs use feature extraction, reversible transformation [2], or other technologies to recognize face images after deformation, but the recognition accuracy is often not ideal due to the influence of the original image deformation. In addition, there are some PPTs that can protect face privacy in public databases, such as deep neural networks that are used to remove soft biometric information [3] such as gender, age, identity, etc. Although face images processed by such methods can achieve a certain degree of privacy protection, they cannot be used directly for face recognition.

A clear and complete face image is not necessary in face recognition. Only parts of the high-order and local features of the face are retained, which can also be used for the purpose of achieving face recognition. For example, eigenfaces can be recognized by using PCA (principal component analysis) to propose partial features of the face. However, the eigenface recognition accuracy proposed by PCA is not high, the accuracy is less than 99%, and the privacy-protection effect is not ideal. On this basis, this paper proposes a privacy-preserving face recognition method based on randomization and local feature learning (RLLFPR), which combines privacy protection and recognition to create a fuzzy face to protect privacy. The algorithm uses a deep neural network with random convolution and self-learning batch normalization layers and combines a self-learning update parameter network with a loss-function backpropagation update parameter network. In this process, the fuzziness, revocability, and irreversibility of the privacy preserving of faces are guaranteed. The network randomizes the pixel values in the facial image and maintains the light-and-dark relationship between the pixel values in the region so that the biometric characteristics of the generated facial image cannot be recognized by human vision; this process is irreversible and can only be recognized by the deep neural network model with specific parameters. At the same time, the deep neural network model cannot infer the original face. The contributions of this paper are as follows:

- We deeply study the learning of hidden human visual information in the end-to-end face image feature learning of deep neural networks and propose that preserving the light-and-dark relationship between facial image pixels and randomizing other information can eliminate human visual information while maintaining the recognizability of facial images. According to the results of our review, the proposed method is the most thorough method to eliminate human visual information in the current privacy-preserving face recognition methods. Using this technology can make a facial image have better privacy-protection ability.
- A deep neural network framework and RLLFPR method for face privacy protection are proposed. Different from traditional encryption and decryption methods, the proposed framework combines face privacy protection with face recognition optimization, which can jointly compute face privacy protection and recognition.
- The RLLFPR method produces privacy-preserved faces with fuzziness, revocability, and irreversibility for better privacy protection. All the information stored in the face recognition server, private face recognition model, private facial image, etc., cannot restore or deduce the original face, which improves the privacy protection of facial images in face recognition or authentication systems.

## 2. Related Work

### 2.1. Face Recognition

Deep neural networks perform well in recognition and classification tasks [4]. The deep neural network feature vector can effectively close and open the intraclass distances during face recognition. Schroff [5] proposed the FaceNet algorithm and employed a triple-loss method to design the loss functions. FaceNet expanded the class distances more than the traditional Softmax method [6]. However, triplet-loss training was challenging on large datasets. Wen [7] proposed the center-loss method to enhance Softmax’s recognition ability. It is hoped that the sum of the distance between all the image features of a class and the center features of that class is minimized. Liu proposed the Sphereface method [8] and introduced angle boundaries into Softmax to improve the face recognition accuracy. The complex-loss-function training led to network training instability. Wang proposed the Cosface method [9] to increase the target’s cosine angle penalty. Cosface exhibited better performance and was easier to implement than Sphereface. Deng proposed the ArcFace method [10], with an angular margin penalty added to the cosine angle. The ArcFace method exhibited compact class arrangement and higher discrimination, which resulted in further improvements.

### 2.2. Face Privacy Protection

Face privacy preservation [11,12] is also an important issue arising with the development of deep neural networks and face recognition. Face data are directly related to personal property and are widely valued by researchers. Traditional face privacy protection is achieved by blurring or pixelizing the main area of the face [13], but blurring may lead to the loss of other information except the face information. Recently, deep neural networks have also been widely used in face privacy-protection technology. In order to protect face data in public databases, the use face-swapping technology or face-derecognition technology are good methods, and most of these technologies generate new faces with the help of GAN [14] technology. Face-swapping technology aims to replace the face in the original image with other faces to achieve image privacy protection. Korshunova [15] used a patch-based style loss function and a variety of style images to quickly change faces. Yang [16] realized the face privacy-protection method of face swapping based on a reversible mask network, which generated more realistic face images and had reversibility. Face-swapping technology has a good effect on face privacy protection, but it cannot be applied to face recognition. Face derecognition has been more widely studied than face-swapping technology. People try to remove soft biometric information such as gender and age from face images without changing the original face. Mirjalili [17] proposed PrivacyNet to generate face images that can interfere with race, gender, and age through a GAN model based on consistency loss. Liu [18] proposed an attribute-preserving face derecognition framework, which can discard some privacy attributes while retaining the required facial attributes. Face-derecognition technology applied in the field of face recognition can only play an auxiliary role in face recognition because the deletion of specific soft biometric information will lead to a reduction in accuracy.

### 2.3. Privacy-Protected Face Recognition

Several privacy-protection technologies exist in face recognition [19]. Recognition by facial encryption and decryption [20] is a fundamental method in this regard. Bai [21] proposed a privacy-preserving face recognition system using homomorphic encryption and multiparty secure computation. Ma [22] proposed a lightweight privacy-preserving adaptive-enhancement (AdaBoost) face recognition framework based on encrypted sharing and edge computing. Boragule [23] stored facial features in smart card memory to realize portable and privacy-preserving recognition. Im [24] proposed the encryption of the feature vectors after deep neural network processing but not of the images. The face recognition privacy was protected by storing the encrypted feature vectors on the server. Zhang [25] proposed a secure and efficient outsourcing protocol for face recognition based on PCA. Through the transformation of the original image information, the information privacy was protected and the resources were saved. Lei [26] proposed a new secure inner product protocol that used a lightweight random mask technique instead of time-consuming public key cryptographic operations to efficiently measure the similarity of facial data. Mai [27] generated reversible random secure sketches through facial feature maps to protect irreversible and deletable templates while maintaining verification performance. The privacy-protection methods of cryptography often need to decrypt and restore the original face for recognition, which has certain security risks. At the same time, the existence of the key is also a burden to the holder. Therefore, these methods are mostly used for face authentication rather than large-scale face recognition. You [13] proposed a reversible privacy-preserving facial expression recognition method in which face information was hidden in mosaics through an adversarial method. However, privacy-preserved images can only complete expression recognition. Walia [28] used the adaptive weighted graph approach to generate multimodal cancelable biometric templates. The multimodal approach increases the complexity of the overall system. Morales [29] used deep neural networks to suppress sensitive information from faces while ensuring correct recognition. Zhang [30] generated perturbed images with hidden attributes while retaining the effect of face verification. Refs. [29,30] decreased the accuracy during face recognition due to the suppression of soft biometric information in the face image. Wu et al. [31,32] proposed the technical idea of using biometric key technology to directly generate a strong biometric key from the biometric characteristics of the client, which means the server does not need to save the biometric template, so as to protect privacy. At present, the stability of the key generated by this technical route needs to be improved. It is difficult to directly blur the original face for face recognition. The existing methods have poor recognition accuracy or privacy-protection effects. By using local differential privacy, Chamikara [33] used differential privacy to apply disturbances to eigenfaces. The privacy-protection effect was better, and the screen was human vision. However, the accuracy was low, only 80%–90%. Zhang [2] achieved the purpose of privacy protection by using an Arnold transformation to process faces and recognize the transformed faces. However, the recognition accuracy of the method needs to be improved. Mi [34] adopted frequency domain collaborative reasoning and proposed DuetFace. The accuracy of the privacy-preserving face recognition was greater than 99%, but the privacy-preserved face retained part of the original face contour. Subsequently, Mi [35] proposed the random selection of frequency components for training and learning, and the recognition accuracy was further improved, but the privacy-protected face still retained the original human part of the information and did not completely shield the image from human vision.

## 3. Privacy-Preserving Face Recognition Based on Randomization and Local Feature Learning (RLLFPR)

In face recognition, it is not necessary to learn the whole face, only the difference of the pixel values of each organ of the original face so that the face can be accurately recognized. Based on the above observations, this paper proposes the RLLFPR framework and method to recognize faces by randomizing the pixel values in the facial image and maintaining the light-and-dark relationship between the pixel values in the facial image. At the same time, the facial image cannot be recognized by the human eye, and the process is irreversible.

**Figure 1:**
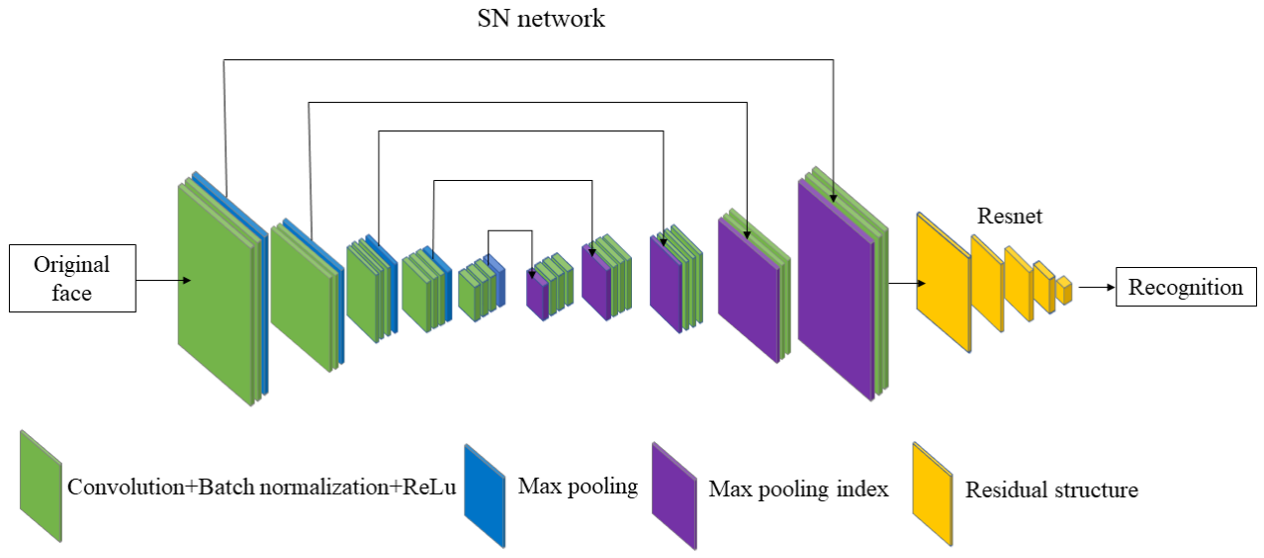
Privacy-preserving face recognition based on randomization and local feature learning (RLLFPR) structure.

RLLFPR consists of two main networks, the SN network (represented by the blue–green–purple parts in Figure 1) and the ResNet [36] network (represented by the yellow part in Figure 1). The SN network is responsible for generating privacy-preserved faces, while the ResNet network is used for recognizing these privacy-preserved faces. The SN uses SegNet [37] as the main module, and the SegNet network is an encoder–decoder network. The max-pooling layer is used for downsampling in the encoder network. In the decoder network, SegNet is different from previous upsampling methods (such as deconvolution or linear interpolation). It uses the max-pooling layer index generated by the downsampling max-pooling process for upsampling. The concrete SN adopts a five-layer encoder–decoder structure and removes the last Softmax layer, and the number of output channels of the last convolutional layer is one. The green part is composed of three parts: convolutional layer + batch normalization layer + ReLU activation function. The blue part is the max-pooling layer, which not only is used in the downsampling but also produces the max-pooling index (the purple part in Figure 1) for upsampling.

### 3.1. Privacy-Preserving Face Recognition Based on Randomization and Local Feature Learning (RLLFPR) Training Method

**Figure 2:**
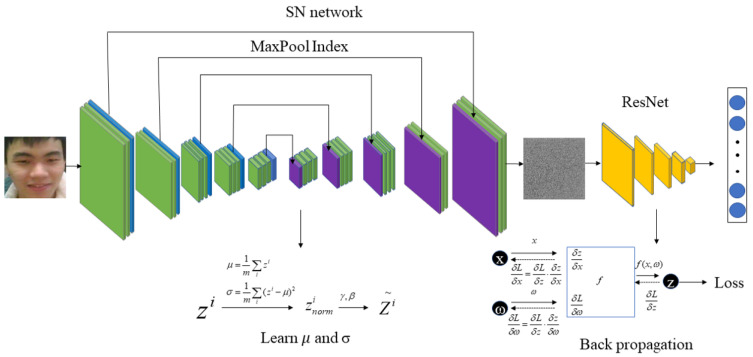
Training process of privacy-preserving face recognition based on randomization and local feature learning (RLLFPR).

**Figure 3:**
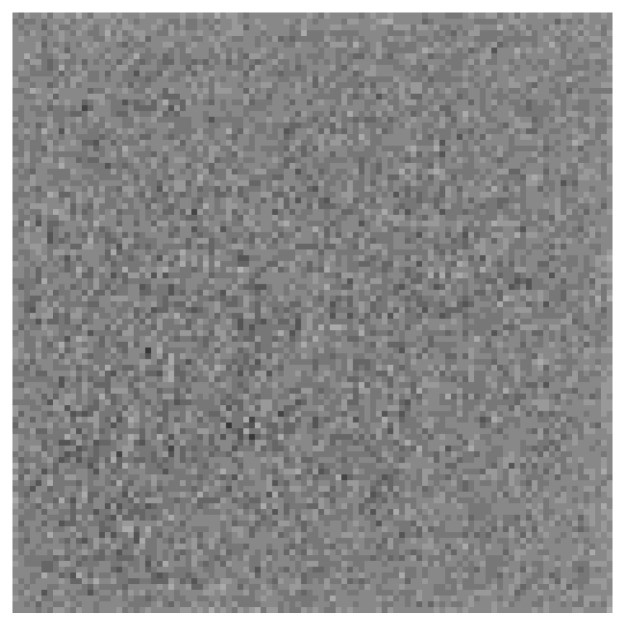
The original face is passed through the SN network to produce the privacy-preserved face, the SN_PrivacyFace (SNPF).

During the training process of the RLLFPR, although the two networks are jointly trained, the backpropagation of the loss function only involves the ResNet network and does not encompass the entire network. The SN network learns through batch normalization layers, enabling self-learning. Figure 2 illustrates the changes in facial images during training and how the parameters of the two networks are updated. The SN network learns through the batch normalization layers and generates SN_PrivacyFace (SNPF) images that are difficult for human vision to recognize, as depicted in Figure 3. The ResNet parameters are learned through the backpropagation of the Cosface loss function (Equation (1)). After training, the ResNet network can accurately recognize SNPF images.

(1) $$Loss=\frac{1}{N_{i}}\sum_{i}-\log\frac{e^{s\left(\cos\left(\theta_{y_{i}},i\right))-m\right)}}{e^{s\left(\cos\left(\theta_{y_{i}},i\right))-m\right)}+\sum_{j\neq y_{i}}e^{s\left(\cos\left(\theta_{y_{i}},i\right)\right)}}$$

### 3.2. Randomized Convolution and Batch Normalization Learning for RLLFPR

**Figure 4:**
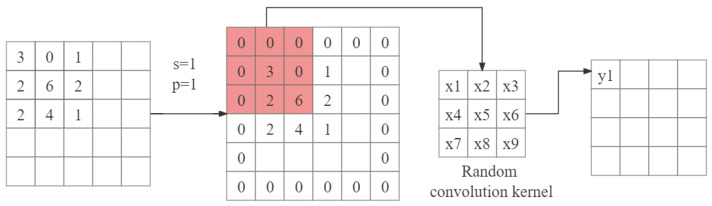
Randomized convolution operation.

In RLLFPR, the SN utilizes random convolution through the convolutional layers to randomize the pixel values of the face. The batch normalization layer, with learned mean and variance, preserves the brightness relationship between the pixel values within the face region. During the initial training phase, the SN randomly initializes the convolutional kernels in all the convolutional layers used in the upsampling and downsampling processes of the network structure. The values of the convolutional kernels follow a normal distribution. Without backpropagation of a loss function, the parameters of the convolutional layers remain unchanged, and the convolutional kernels retain their initial random values. The convolution operation process is shown in Figure 4, where x1–x9 are the random values of the initial convolution kernel.

In the SN, images after multiple rounds of random convolution operations can have high fuzziness. In the convolution operation, it is assumed that the original image is a grayscale image of n × n (n is even). Equation (2) is the output image size formula of randomized convolution; input, *p*, *k*, *s*, and output represent the input image size, zero filling, convolutional kernel size, stride, and output image size, respectively. After the convolution operation with the convolution kernel size *k* = 3, stride *s* = 1, and zero filling *p* = 1, the image size is still n × n (Equations (2) and (3)). In this process, the pixel values in the image are randomized to generate privacy-protected images that are difficult for human vision to recognize.

(2) $$ouput=\lfloor \frac{input+2p-k}{s}\rfloor +1$$

(3) $$\begin{array}{c} {\left(\begin{array}{ccccc} a_{11} & a_{12} & \ldots & a_{1(n-1)} & a_{1n}\\ a_{21} & a_{22} & \cdots & a_{2(n-1)} & a_{2n}\\ \vdots & \vdots & \ddots & \vdots & \vdots \\ a_{(n-1)1} & a_{(n-1)2} & \cdots & a_{\left(n-1)(n-1\right)} & a_{(n-1)n}\\ a_{n1} & a_{n2} & \cdots & a_{n(n-1)} & a_{nn} \end{array}\right)}_{n\times n}⊗k\overset{s=1}{⟶}{\left(\begin{array}{ccccc} b_{11} & b_{12} & \ldots & b_{1(n-1)} & b_{1n}\\ b_{21} & b_{22} & \cdots & b_{2(n-1)} & b_{2n}\\ \vdots & \vdots & \ddots & \vdots & \vdots \\ b_{(n-1)1} & a_{(n-1)2} & \cdots & b_{\left(n-1)(n-1\right)} & b_{(n-1)n}\\ b_{n1} & b_{n2} & \cdots & b_{n(n-1)} & b_{nn} \end{array}\right)}_{n\times n} \end{array}$$

The SN network first reduces and then enlarges the facial image, with the downsampling process primarily utilizing the max-pooling layer. The max-pooling layer is similar to a convolutional layer but does not require convolutional kernels. It is used to reduce the size of the feature map and extract the main features. Specifically, the max-pooling operation takes the maximum value within each region and assigns it the value at the corresponding position in the output feature map, as shown in Equation (4). In other words, it summarizes the pixel values within each region by taking the maximum value. The output feature map has a reduced size but retains the most significant features within each region. The output image size is determined by Equation (2), where *k* represents the region size, *s* is the stride, and *p* is the zero padding. Additionally, during the downsampling process, the position indices of the max-pooling layer are saved and used for upsampling in subsequent steps.

(4) $${\left(\begin{array}{ccccc} a_{11} & a_{12} & \ldots & a_{1(n-1)} & a_{1n}\\ a_{21} & a_{22} & \cdots & a_{2(n-1)} & a_{2n}\\ \vdots & \vdots & \ddots & \vdots & \vdots \\ a_{(n-1)1} & a_{(n-1)2} & \cdots & a_{\left(n-1)(n-1\right)} & a_{(n-1)n}\\ a_{n1} & a_{n2} & \cdots & a_{n(n-1)} & a_{nn} \end{array}\right)}_{n\times n}\overset{k=2,s=2,p=0}{⟶}{\left(\begin{array}{ccc} a_{11} & \ldots & a_{1n}\\ \vdots & \ddots & \vdots \\ a_{n1} & \cdots & a_{nn} \end{array}\right)}_{\frac{n}{2}\times \frac{n}{2}}$$

During the upsampling process, the downsampling max-pooling layer’s index matrix is utilized to enlarge the reduced image. The maximum values obtained from the downsampling process are placed back into their original positions in the matrix using the index positions, while the other values within the region are set to 0. This process is described by Equation (5), where *k* represents the region size, *s* is the stride, and index refers to the index matrix. By performing upsampling the same number of times as downsampling, the image can be restored to its original size. Utilizing the max-pooling layer’s index to upscale the image reduces the number of network parameters and improves computational efficiency.

(5) $${\left(\begin{array}{ccc} a_{11} & \ldots & a_{1n}\\ \vdots & \ddots & \vdots \\ a_{n1} & \cdots & a_{nn} \end{array}\right)}_{\frac{n}{2}\times \frac{n}{2}}\overset{k=2,s=2,index}{⟶}{\left(\begin{array}{ccccc} a_{11} & 0 & \ldots & 0 & a_{1n}\\ 0 & 0 & \cdots & 0 & 0\\ \vdots & \vdots & \ddots & \vdots & \vdots \\ 0 & 0 & \cdots & 0 & 0\\ a_{n1} & 0 & \cdots & 0 & a_{nn} \end{array}\right)}_{n\times n}$$

During the downsampling process in the SN network, randomization is introduced through convolutional layers, adding randomness to the image. The image size is reduced and some information is removed through the max-pooling layer. The removed information cannot be fully recovered during the unlearned upsampling process, resulting in differences between the generated image and the original image. The combination of randomness and removed information creates privacy-preserved images that are difficult for human vision to recognize.

**Figure 5:**
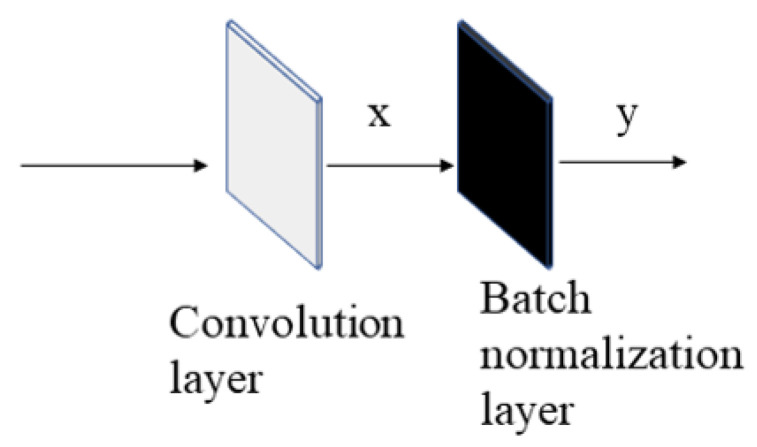
Convolutional layer and batch normalization layer in SN network.

The convolution operation adds randomness while preserving some information from the original image. This information is enhanced in the batch normalization layer. The self-learning of the batch normalization layer can learn the light-and-dark difference of pixel values in the image, which can be recognized by the subsequent network. The batch normalization layer specifically learns the mean and variance of the data. Different from the convolutional layer, the mean and variance of the batch normalization layer in the training mode can also update the parameters without loss-function backpropagation. The SN structure has a convolutional layer followed by a batch normalization layer (Figure 5). x is the value after the randomized convolutional layer, and y is the value of x after the batch normalization layer; the mean(x), Var(x), represented mean, variance, and eps prevent the divisor from being zero. λ and β represent the updated backpropagation parameters, defaulting to 1 and 0 (Equation (6)). On the basis of the preset hyperparameter m, the variance and mean will constantly self-learn and update during the training process (Equations (7) and (8)). x and x’ denote different batches of data. In the prediction stage, the batch normalization layer processes the data with the mean and variance learned by training, and no more updates are performed.

(6) $$y=\frac{x-mean(x)}{\sqrt{Var(x)}+eps}\gamma +\beta$$

(7) $$mean=\left(1-m\right)*mean\left(x\right)+m*mean\left(x^{\prime }\right)Var=\left(1-m\right)*Var\left(x\right)+m*Var\left(x^{\prime }\right)$$

(8) $$Var=\left(1-m\right)*Var\left(x\right)+m*Var\left(x^{\prime }\right)$$

**Figure 6:**
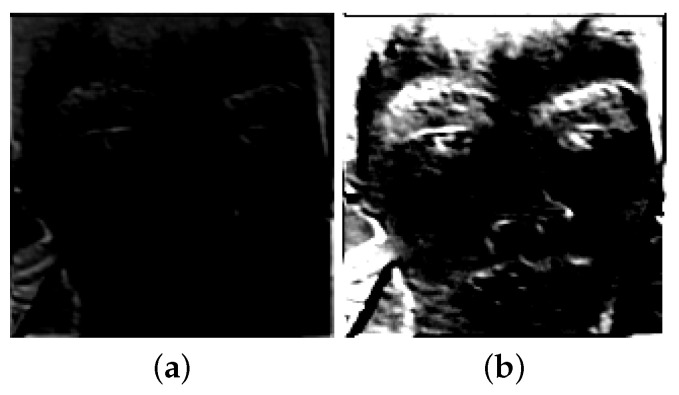
(**a**) Facial image after the convolutional layer processing; (**b**) facial image after the convolutional and batch normalization layer processing.

Since the convolution kernel is random, the image is passed through a convolution layer to generate a lower-information image (Figure 6a). After multiple convolutional layers, affected by the randomized convolution kernel, the image has no information. Such images cannot be used for face recognition. However, we observe that images combined by convolutional layers and batch normalization layers can generate preliminarily recognizable facial images (Figure 6b). The facial image in Figure 6b contains more recognition features, such as face contour, eye, nose, mouth, and other contours, and other original image information than the facial image in Figure 6a. The whole process is as follows: the convolution layer randomizes the pixel values of the facial image in the region, and the batch normalization layer maintains the light-and-dark relationship between the pixel values in the region. After multiple rounds of this cycle, the privacy-protected face image that is difficult to recognize by human vision but can be accurately recognized by machine vision is generated.

In the convolution process, the randomness of the convolution kernel also makes the final image revocable and irreversible. The randomness of the convolution kernel makes the same image obtain different results through different convolution kernels, see Equation (3), and this randomness will also be enhanced in multiple convolution operations. Different random convolution kernels are used for each training, so the networks between training cannot communicate with each other. This makes the facial images in the database revocable, and even if the server-side face data are stolen (the server-side stores the privacy-preserved facial images), the whole network can be retrained so that the stolen facial images cannot be recognized normally. At the same time, the convolution operation is irreversible, so the resulting image is also irreversible.

## 4. Experiment

### 4.1. Setting

We used RLLFPR with the LFW (Labeled Faces in the Wild) [38], Celeba, and HDU (camera-captured facial images) datasets. The LFW dataset consists of 13,233 face images. The celebrity dataset Celeba randomly selects 10% of its face images (20,177 faces) for training and testing. The HDU dataset collected by our camera consists of 3891 face images of 51 young people (around 20 years old) under different background and lighting conditions. These faces were uniformly aligned and enlarged to 160 × 160 size by the MTCNN [39] method. The ResNet network was updated to ResNet50. The loss function used Cosface (S = 30, M = 0.5) (Equation (1)). The Python language, Pyotch1.8 library, and GTX 2080TI graphics card were used. A Microsoft LifeCam HD-3000 was used to capture the images.

The ResNet50 was first pretrained using the LFW dataset with epoch = 256, learning rate = 0.1, and batchsize = 32. After pretraining the ResNet50, the RLLFPR network was trained, and the SN and ResNet were jointly trained and tested on the training samples. Furthermore, we used Equation (1) as the loss function, epoch = 64, learning rate = 0.001, batchsize = 32.

### 4.2. The Recognition Performance of Privacy-Preserving Face Recognition Based on Randomization and Local Feature Learning (RLLFPR)

We mainly compared the accuracy, misidentification rate, and F1 score of an RLLFPR image with the original face image without privacy protection and images processed with Arnold transform [2], eigenface [40], differential privacy [33], and PartialFace [35] methods on the three datasets, and the specific results are shown in Table 1. The Arnold transform method processes the face through the method of data processing. The eigenface method uses PCA to extract part of the features of the face for recognition. The differential privacy method adds differential privacy on the basis of the eigenface. PartialFace blurs the face by removing the low-frequency information of the image and partially randomizes the high-frequency information.

The original face images without privacy protection, shown in Table 1, performs the best on the three datasets. The privacy-protected faces generated by the AES encryption method cannot be recognized in the case of ciphertext, so the accuracy is about 0 in the tests on the three datasets. On the LFW dataset, RLLFPR had the highest accuracy of 99.93%. It was better than the 99.82% of the Arnold transform method, 83.73% of differential privacy, and 99.80% of PartialFace. In terms of F1 score, RLLFPR was the same as PartialFace, with 99.67%, which was better than the other privacy-preserving recognition algorithms. The misidentification-rate score of RLLFPR was 0.13%, which was slightly worse than Arnold’s 0.10% but better than the other methods. On the Celeba dataset, RLLFPR had the highest recognition accuracy of 98.77%, the highest F1 score of 98.67%, and the lowest misidentification rate of 0.11%, which was better than the existing methods. In the dataset collected by the camera, due to the small size of the dataset and the environment, the lighting and other factors changed greatly. The method of differential privacy performed poorly on the HDU dataset, with an accuracy and F1 score below 50% and misidentification rate greater than 50%. The accuracy of the eigenface method was only 80.39%, and the accuracy of the Arnold transform was only 90.57%. PartialFace and RLLFPR also had excellent results on the HDU dataset, with an accuracy of more than 99%. RLLFPR was better than PartialFace, with an accuracy of 99.58%, misidentification rate of only 0.59%, and F1 score of 99.23%.

Discussion 1: Compared with the previous privacy-protection methods, RLLFPR had a higher accuracy and F1 score and lower misidentification rate. At the same time, compared with the original face image without protection, the accuracy of RLLFPR only had a slight decrease, which was only 0.02% lower on the LFW dataset. On the Celeba dataset, the maximum decline was 0.92%, and the final accuracy was 98.77%, which was also better than the other privacy protection–recognition methods. On the HDU dataset, the accuracy was reduced by 0.39%, and the accuracy was still more than 99.5%. In general, RLLFPR is better than the previous methods in balancing privacy protection and face recognition and is superior to the existing methods.

### 4.3. RLLFPR Fuzzing Performance Test

**Figure 7:**
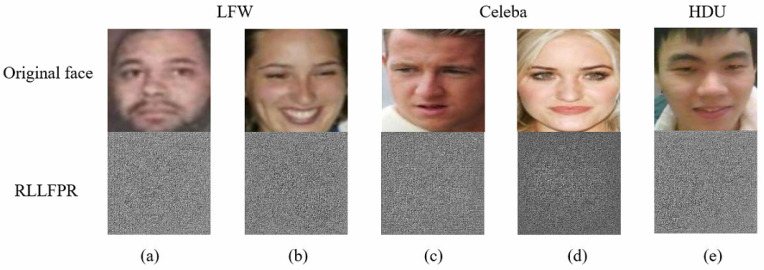
Privacy-preserved faces produced by RLLFPR on different datasets: (**a**,**b**) from the LFW dataset, (**c**,**d**) from the Celeba dataset, and (**e**) from the HDU dataset.

Some privacy-preserved faces produced by RLLFPR in different datasets are shown in Figure 7. Compared with the original faces, the privacy-protected faces generated by RLLFPR in the three datasets changed greatly. From the perspective of human vision, information about the original face cannot be recognized from the privacy-preserved face.

#### 4.3.1. Fuzziness Test Method

In terms of the fuzziness of privacy-protected images generated, because PartialFace retains part of the contours of the face, the recognition accuracy of the differential privacy method was less than 90%. Therefore, the main comparison is between RLLFPR and the Arnold transform method and privacy-protected images produced by AES encryption. RLLFPR, Arnold transform, and AES encryption methods all blur the original face to a large extent, which makes it difficult to identify with human vision.

**Figure 8:**
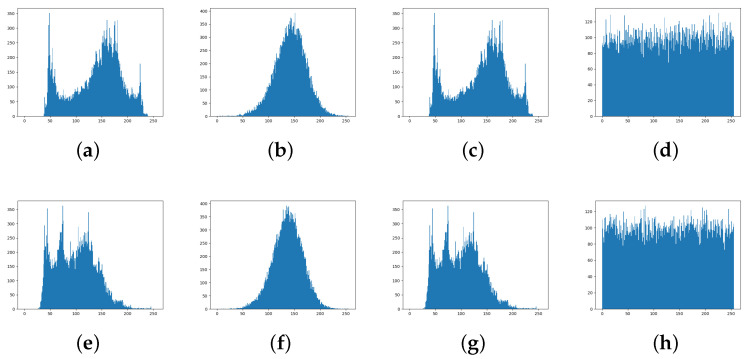
Gray histograms of two different face images from different methods: (**a**,**e**) derived from the original face images, (**b**,**f**) derived from RLLFPR after processing, (**c**,**g**) derived from Arnold transformed images, and (**d**,**h**) derived from AES encrypted images. (**a**–**d**) each represent a human face and (**e**–**h**) represent a different face from (**a**–**d**).

The privacy-preserved faces generated by RLLFPR cannot be recognized by human vision, and some statistical characteristics of their performance are shown in Figure 8. Figure 8 shows the grayscale histogram of the original image, processed by the RLLFPR method, Arnold transform method, and AES encryption method. Compared with the original image (Figure 8a,e), the gray distribution of the Arnold transform image (Figure 8c,g) is basically unchanged, which is related to the restoration of the original image after multiple rounds of operation. The gray histogram obtained by the RLLFPR method (Figure 8b,f) and the AES encrypted image (Figure 8d,h) show similar distributions for different faces; the RLLFPR method shows a similar bell-shaped distribution; and AES shows a similar white-noise distribution. The similar gray histogram distribution of different faces can improve the blurring performance of facial images.

**Figure 9:**
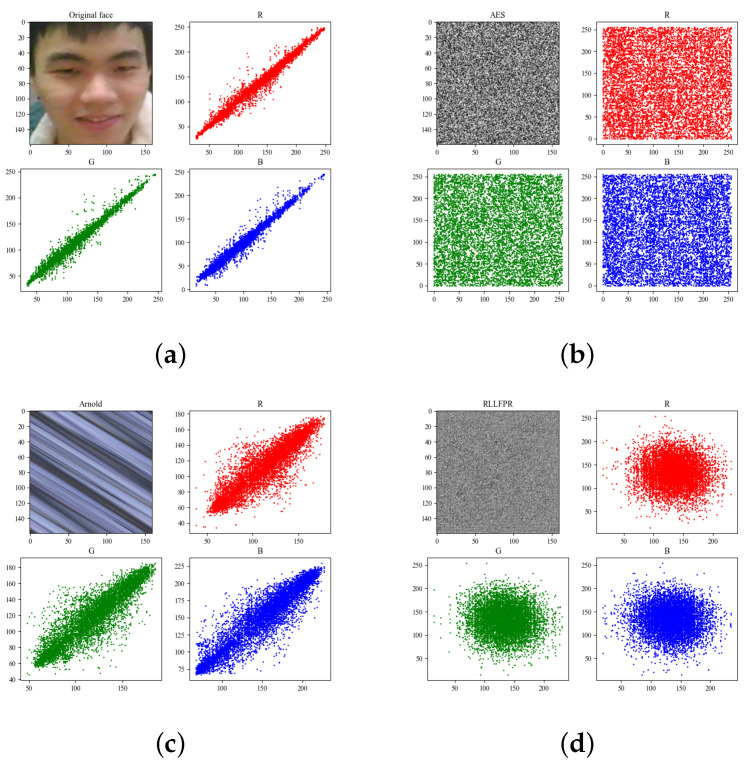
Correlation analysis of adjacent pixels of original face, AES, Arnold, and RLLFPR images: (**a**) original face, (**b**) AES, (**c**) Arnold, and (**d**) RLLFPR.

Figure 9 and Table 2 show the original image, Arnold, AES, as well as RLLFPR image-adjacent pixel correlation analysis. Specifically, 3000 pairs of adjacent pixels from the three R, G, B channels were selected for analysis. As can be seen in Table 2, the original image (Figure 9a) presented a high linear correlation, and the three directional correlation values were all greater than 0.98. Although Arnold transform (Figure 9c) blurred what can be seen with human vision, it only moved pixels between locations, so it still had high correlation, and the three directional phase property values were around 0.8. RLLFPR (Figure 9d) and AES (Figure 9b) showed low correlation in the correlation analysis; RLLFPR showed three correlations around 0.05, and AES had even lower values, around 0.005. Low correlation can indicate that the image pixels are less regular and difficult to predict. In general, the correlation effect of RLLFPR is much higher than that of the Arnold transform and slightly inferior to the AES method.

Table 3 shows a comparison of the PSNR and UACI values of the privacy-protected face images generated by RLLFPR with the Arnold transformed, noisy, and AES encrypted images. The noise-processed face is still recognizable and can be used as an intermediate value for comparison. The PSNR value is the highest and the UACI value is the lowest for the face after noise processing, indicating its high recognizability. The processed image with noise has less distortion and blurring. The RLLFPR, Arnold, and AES encrypted versions of all three images are not visible to the human eye and show better results in terms of PSNR and UACI. The RLLFPR method is similar to the Arnold transform in terms of both data PSNR and UACI, with PSNR values of 11.80 and 11.53 and UACI values of 50.61 and 53.93. All methods resulted in an AES-encrypted ciphertext with values of 8.77 and 75.27. However, AES-encrypted images cannot be recognized.

In addition, we designed three fuzziness tests.

**Figure 10:**
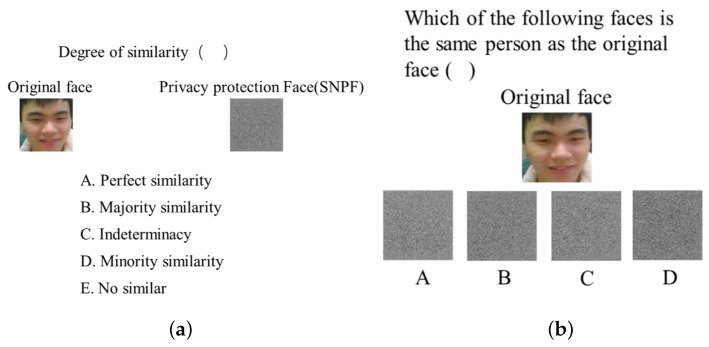
Composition of questionnaire questions. (**a**) the direct similarity between the privacy-protected image and the original image; (**b**) the direct matching degree between the privacy-protected image and the original image.

First, we conducted human evaluation experiments by means of questionnaires. The questionnaire consisted of two types of single-choice questions. The experimental group was based on the privacy-preserved faces generated by RLLFPR, and the control group comprised the privacy-preserved images processed by AES encryption. The first single-item choice was entitled “Similarity problem” (Figure 10a), which judged the degree of similarity between two faces by giving the original facial image and the privacy-preserved image of the original face. Five options were given, respectively, is completely not similar (4 points), a small degree of similarity (3 points), not sure (2 points), most similar (1 points), and completely similar (0 points), using scoring judgment of fuzzy degrees. The higher the score, the higher the fuzziness of the image.

The second type of single-choice question (Figure 10b) was a matching question. On the basis of the original face, four faces after privacy protection were given, and the privacy-protected face that the participant believed belongs to the same person as the original face was selected. The lower the accuracy, the higher the image fuzziness.

A total of 100 questionnaires (groups 1 and 2 each had 10 questions) were distributed, and 98 were collected, resulting in an effective response rate of 98%. The survey results are considered valid. The statistical results for each question in the questionnaire are as follows (Table 4).

Discussion 2: The statistical results for each question in the questionnaire are as follows (Table 4). It can be observed that the privacy-preserved face generated by RLLFPR (SNPF) and the face encrypted with AES show similar results. Both methods received high scores in the first group. The similarity values are 3.51 and 3.55, respectively, which reflects that the privacy-preserved images generated by RLLFPR are visually similar to the effect of the AES-encrypted images and that the processed and original images have a low similarity. In the second group, the matching accuracy is close to 25%, indicating that the respondents were close to randomly selecting their answers. Accuracy close to 25% proves that both images could not be matched to the original image, and even if privacy-preserved images were acquired, they could not be matched to a specific person.The results of the two questionnaires show that the privacy-preserved faces generated by RLLFPR have high ambiguity, similar to encryption methods. PartialFace [35] achieved high similarity compared to the original face in the first questionnaire, and in the second questionnaire, humans recognized privacy-protected faces with accuracies reaching or even exceeding 70–80%. Therefore, it was judged that the PartialFace method for human eye visual blurring was unsatisfactory, and no subsequent blurring test was performed.

Second, the idea of convolution is used to calculate the mean value of the image block, and the variance (Equations (9)–(11)) is calculated for all the mean values. s is the block size, which means the mean value of the s × s region size. Sharp images are colorful, and V(img) tends to be larger, while fuzzy images are smaller. $\frac{V(img_{i})}{V(img_{o})}$ is generally between 0 and 1. $img_{i}$ refers to privacy-protected images and $img_{o}$ refers to original images:

(9) $$V(img)=\mathrm{var}(img_{i}⊗k_{s\times s})$$

(10) $$k_{s\times s}=\left(\begin{array}{ccc} \frac{1}{s*s} & \ldots & \frac{1}{s*s}\\ \vdots & \ddots & \vdots \\ \frac{1}{s*s} & \cdots & \frac{1}{s*s} \end{array}\right)\;$$

Fuzziness 2 is shown in Equation (11); the larger the fuzziness of the image, the higher the value.

(11) $$Fuzziness2=1-\frac{V(img_{i})}{V(img_{o})}$$

The third kind of fuzziness is the method of edge detection (Equations (12) and (13)) + SSIM similarity detection (Equation (14)). Firstly, the edge of the image is detected, and then the SSIM similarity between the privacy-protected image and the original image is calculated. The lower the similarity, the higher the ambiguity. The Laplacian operator is used for edge detection.

(12) $$Laplacian\left(img\right)=\frac{\partial^{2}img}{\partial x^{2}}+\frac{\partial^{2}img}{\partial y^{2}}$$

(13) $$k_{laplacian}=\left(\begin{array}{ccc} 0 & -1 & 0\\ -1 & 4 & -1\\ 0 & -1 & 0 \end{array}\right)$$

The SSIM algorithm [41] similarity index measures the similarity of images, ranging from 0 to 1. The larger the value, the higher the similarity of the image; μ and σ represent the mean and standard deviation, respectively.

(14) $$SSIM\left(x,y\right)=\frac{\left(2\mu_{x}\mu_{y}+c_{1}\right)\left(2\mu_{xy}+c_{2}\right)}{\left(\mu_{x}^{2}+\mu_{y}^{2}+c_{1}\right)\left(\sigma_{x}^{2}+\sigma_{y}^{2}+c_{12}\right)}$$

The image fuzzy degree Fuzziness3 is obtained by subtracting the SSIM similarity from 1 (Equation (15)), and the higher the value, the higher the fuzzy degree.

(15) $$\begin{array}{c} Fuzziness3=1-SSIM(lap\left(img_{o}\right),lap\left(img_{i}\right)) \end{array}\;$$

#### 4.3.2. Fuzzy Performance of Privacy-Preserving Face Recognition Based on Randomization and Local Feature Learning (RLLFPR)

Table 5 shows the values obtained by different privacy-protection methods after the fuzziness test methods, and the comparison of privacy-protection performance between different methods is added in Table 5. The Fuzziness1 results are shown in Table 4. As can be seen from the table, the ambiguity of RLLFPR measured by Fuzziness2 is in the range of 0.80–0.95, which is obviously better than that of noise addition, Arnold transformation, and other methods. AES is the encryption algorithm with ambiguity in the range 0.6–0.9, and the encrypted image cannot be directly recognized. Measured by Fuzziness3, RLLFPR also has a good effect. RLLFPR (0.997) is better than Arnold (0.996), and noise (0.992) is second to AES (0.999). In summary, the privacy-preserving facial fuzzy image generated by RLLFPR is better than the additive noise method and Arnold method and is similar to the AES encryption method. The generated faces in RLLFPR can effectively obscure human vision, providing protection against unauthorized access to facial data stored on servers. RLLFPR eliminates the need for encryption keys, reducing the burden on individuals concerned about their identification. In addition, RLLFPR can directly recognize privacy-protected images.

Discussion 3: In terms of security, RLLFPR is irreversible as well as revocable. Irreversibility: The core of the SN network is composed of a convolutional layer, a batch normalization layer, and an activation-function and maximum-pooling layer. The convolution operation in the convolutional layer and the pooling operation in the maximum-pooling layer are irreversible operations. The activation function uses a nonlinear ReLU activation function, which is also irreversible. Therefore, with the stacked use of irreversible operations, an irreversible privacy-protected image will eventually be produced, and even if the privacy-protected image is obtained, the probability of recovering from it the original face image is close to 0. The ciphertext encrypted by AES cannot be recognized directly, and the Arnold transformed image can be used to recover the original image after many transformations. This all increases the risk of privacy leakage.

Revocability: Since RLLFPR generates blurred images that cannot be recognized by the human eye, it is impossible to match the face images in the database with the real face images even if the database is stolen. The Arnold transform method is not revocable since its images can be restored to the original images. Combining the two aspects of blurriness and security, RLLFPR is more advantageous than the previous methods.

### 4.4. Network Structure Ablation Experiment of RLLFPR Method

**Figure 11:**
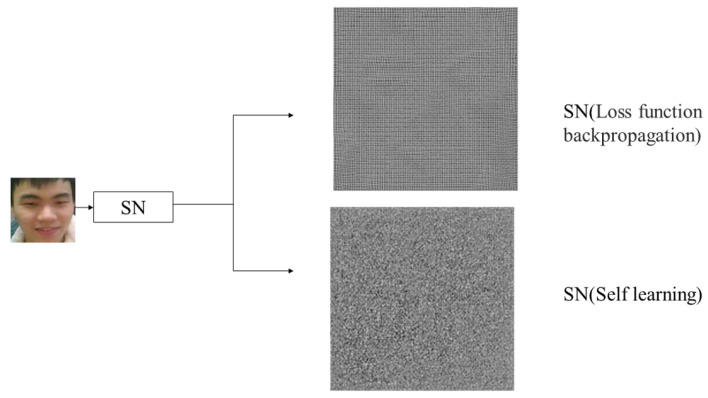
Different privacy-preserved images produced by SN network after self-learning and SN with loss function backpropagation.

During the training process of RLLFPR, the entire SN network does not update its parameters through the loss function. To demonstrate the effectiveness of the structural improvement, experiments were conducted on the SN network with parameter updates through loss backpropagation. In this case, the SN network still used the Cosface loss function (Equation (1)). However, upon careful observation, it was found that the SN network trained with loss backpropagation retained the contour information of the original face, resulting in suboptimal privacy protection (Figure 11, the generated privacy-protected image is enlarged). The experiments tested these two types of images under three different levels of blurriness (Table 6). From Table 6, it can be observed that the facial images generated by the RLLFPR method have higher privacy-protection effectiveness compared to the SN network trained with the loss function. In the first fuzziness test method, the similarity value of SN after self-learning is 3.51, which is higher than that of SN after loss backpropagation, which is 2.11. It is proved that the face image produced by the self-learning SN is shielded from human-eye vision. In the second and third fuzziness test methods, the self-learned SN is 0.80–0.95 and 0.997, respectively, which is better than the SN after loss function backpropagation of 0.75–0.95 and 0.983. Thus, the self-learned SN produces more ambiguous privacy-preserved faces than the SN after loss function backpropagation.

For the recognition of privacy-protected faces on the backbone network, a ResNet was chosen due to its superior performance in face recognition and image classification. Specifically, ResNet50 was selected. Additionally, DenseNet [42] was compared to the ResNet, as it has a similar excellent effect in image classification. For DenseNet, we selected DenseNet121. The results of the training on the LFW dataset are presented in Table 7. Both ResNet50 and DenseNet121 showed similar performance in terms of accuracy, with ResNet50 achieving a slightly higher accuracy of 99.93% compared to DenseNet121’s 99.91%. However, ResNet50 had a higher misidentification rate of 0.13% compared to DenseNet121’s 0.07%. Both models had the same F1 score of 99.67%. Therefore, either backbone network can be selected for DenseNet.

## 5. Conclusions

In this paper, we study the recognizable space of face images and find that under the condition of appropriately maintaining the structure of face components and local interpixel light-and-shadow relations, randomizing the rest of the information can still allow the model to accurately recognize face images with a recognition accuracy of >99%. On this basis, a face privacy recognition method, RLLFPR, is proposed. Instead of using traditional cryptography or image-processing methods to generate privacy-protected faces, RLLFPR takes advantage of the inherent randomness of the convolutional layer and the self-learning function of the batch normalization layer in deep neural networks. By randomizing the pixel values between face image regions and maintaining the light–dark relationship between face pixels, it generates privacy-protected faces that are difficult to recognize by human vision, with a recognition rate of nearly 25% in a four-choice–one-human recognition test, which is equivalent to the recognition rate of random guessing. At present, the RLLFPR method requires a high number of face training sets and mainly needs to adapt to as many lighting environments as possible. Further research considerations include continuing to explore the identifiable space of facial images and building a more portable, controllable, and effective network structure to generate facial images with better recognition effects and privacy-protection effects.

## Figures and Tables

**Table 1:** Comparison of accuracy, misidentification rate, and F1 score of RLLFPR with currently available face privacy-preserving recognition methods on three datasets (Unit/%).

Database			LFW			Celeba			HDU	
Methods	Privacy Protection	Accuracy	Misidentification	F1	Accuracy	Misidentification	F1	Accuracy	Misidentification	F1
Original face	No	99.98	0.10	99.83	99.69	0.08	99.73	99.97	0.10	99.75
AES	Yes	0	∖	∖	0	∖	∖	0	∖	∖
Eigenface [40]	No	98.93	0.45	98.88	98.41	0.52	98.41	80.39	18.90	79.61
Arnold [2]	Yes	99.82	0.10	99.66	98.33	0.15	98.13	90.57	9.06	89.74
Differential	Yes	83.73	8.77	83.61	81.68	9.31	81.98	<50	>50	<50
privacy [33]										
PartialFace [35]	Yes	99.80	0.15	99.67	98.73	0.24	97.91	99.34	0.78	98.94
RLLFPR	Yes	99.93	0.13	99.67	98.77	0.11	98.67	99.58	0.59	99.23

**Table 2:** The average correlation of RGB channels of the original face, AES, Arnold, and RLLFPR images in the horizontal, vertical, and diagonal directions.

Method	Horizontal	Vertical	Diagonal
Original face	0.9923	0.9916	0.9829
Arnold	0.8608	0.7548	0.9279
AES	−0.0039	−0.0032	−0.0007
RLLFPR	−0.0929	0.0326	−0.0795

**Table 3:** Comparison of accuracy and error rates of different methods.

Method	PSNR	UACI
Noise	14.16	36.24
Arnold	11.53	53.93
AES	8.77	75.27
RLLFPR	11.80	50.61

**Table 4:** Questionnaire results.

Purpose of the Question	Types of Design Problems (Two Groups)	Experimental Group	Control Group (AES)
Fuzziness test	Group 1 dissimilarity	3.51	3.55
	(0–4, 0 clear, 4 no similarity)		
	Group 2 match	20.90%	25.35%
	(choose 1 from 4, measure accuracy)		

**Table 5:** Fuzziness and safety analysis of RLLFPR, AES, Arnold, and noise methods.

Method	Privacy Protection	Identification after Protection	Fuzziness2	Fuzziness3	Key	Reversibility	Revocability
Arnold	Yes	Yes	0.40–0.70	0.996	No	Reversible	Revocable
AES	Yes	No	0.60–0.90	0.999	Yes	Reversible	Revocable
Noise	Yes	Yes	0.20–0.40	0.992	No	Irreversible	Irrevocable
Original face	No	∖	0	0	No	∖	Irrevocable
RLLFPR	Yes	Yes	0.80–0.95	0.997	No	Irreversible	Revocable

**Table 6:** Comparison of three blurriness tests for privacy-preserved images produced by SN after self-learning and SN network with loss function backpropagation.

	Fuzziness1	Fuzziness2	Fuzziness3
SN (loss function backpropagation)	2.11	0.75–0.95	0.983
SN (self-learning)	3.51	0.80–0.95	0.997

**Table 7:** Performance comparison of ResNet50 and DenseNet121 as backbone networks in RLLFPR (Unit/%).

	Accuracy	Misidentification Rate	F1
ResNet50	99.93	0.13	99.67
DenseNet121	99.91	0.07	99.67

## Data Availability

The LFW and Celeba face databases used in this paper are publicly available face databases. The images in the HDU face database were captured with the informed consent of the recorded subjects. The HDU database face images disclosed herein have the informed consent of the users. The HDU database is not public.The LFW dataset URL is https://vis-www.cs.umass.edu/lfw/ accessed on 30 January 2024. The Celeba dataset URL is https://mmlab.ie.cuhk.edu.hk/projects/CelebA.html accessed on 30 January 2024.
